# Association between changes in predicted body composition and occurrence of heart failure: a nationwide population study

**DOI:** 10.3389/fendo.2023.1210371

**Published:** 2023-10-23

**Authors:** Ho Geol Woo, Dong-Hyeok Kim, Hyungwoo Lee, Min Kyoung Kang, Tae-Jin Song

**Affiliations:** ^1^ Department of Neurology, Kyung Hee University College of Medicine, Seoul, Republic of Korea; ^2^ Department of Cardiology, Seoul Hospital, Ewha Womans University College of Medicine, Seoul, Republic of Korea; ^3^ Department of Neurology, Seoul Hospital, Ewha Womans University College of Medicine, Seoul, Republic of Korea

**Keywords:** body composition, body mass index, obesity, skeletal mass, fat mass, heart failure

## Abstract

**Background:**

Large population-based studies on the association between changes in body composition and the occurrence of heart failure (HF) are rare. We aimed to determine the association between changes in body composition, including the predicted body fat mass index (pBFMI), predicted appendicular skeletal muscle mass index (pASMI), and predicted lean body mass index (pLBMI), and the occurrence of HF.

**Methods:**

For present study, 2,036,940 people who consecutively underwent national health examinations from 2010~2011 (baseline period) to 2012~2013 (follow-up period) were included. The pBFMI, pASMI, and pLBMI were indirectly investigated using validated anthropometric prediction equations from the Korean National Health and Nutrition Examination Survey cohort. The outcome was defined as at least two or more claims of HF.

**Results:**

During a median of 7.59 years of follow-up, 22,172 participants (event rate, 1.09%) with HF were observed. Decreased changes in the pASMI and pLBMI were associated with the occurrence of HF among males (hazard ratio [HR] 0.966, 95% confidence interval (CI) [0.944-0.988]; HR 0.939, 95%CI [0.923-0.955], respectively) and females (HR 0.924, 95%CI [0.900-0.947]; HR 0.951, 95%CI [0.939-0.963], respectively). An increased change in the pBFMI was associated with the occurrence of HF in males (HR 1.017, 95%CI [1.001-1.034]). However, paradoxically, a change in the pBFMI was associated with the occurrence of HF in females (HR 0.925, 95%CI [0.909-0.942]).

**Conclusion:**

Decreased skeletal muscle mass was related to the occurrence of HF. However, the relationship between a change in fat mass and the occurrence of HF was different and even paradoxical depending on sex.

## 1 Introduction

Heart failure (HF) is a clinical syndrome that is related to decreased cardiac contractility accompanied by impairment of the ejection of blood or ventricular filling (1). HF is a common cardiovascular disease and a leading cause of hospitalization, especially in older adults. The economic burden and prevalence of HF have been increasing worldwide (2). Despite the development of treatment and prevention tools for HF, the morbidity and mortality rates associated with HF are still high (2). Therefore, it is important to identify the hidden risk factors associated with HF. To date, risk factors for HF, including hypertension, diabetes mellitus, coronary artery occlusive disease, aortic atheroma, poor oral hygiene, smoking, and cardiomyopathy, have been suggested. However, information regarding further modifiable associations or occurrence for HF is still lacking (3, 4).

Obesity is defined as an abnormal accumulation of health-impairing fat mass, commonly assessed as a determination of a body mass index (BMI) ≥30 kg/m^2^ (5). The prevalence of obesity has increased over the past few decades throughout the world, and the global burden of obesity is currently increasing rapidly (6). It is well known that obesity, especially greater levels of adiposity, is a well-established risk factor for cardiovascular disease and is associated with cardiovascular risk factors, including hypertension, insulin resistance, lipoprotein metabolism, and inflammation (7, 8). In a previous study, obesity and abdominal fat were found to be associated with an increased incidence of HF (9). However, when obesity was evaluated using BMI in patients with HF, the obesity paradox that obese patients have better long-term and short-term prognosis was observed (10). Furthermore, because BMI includes not only fat mass but also skeletal mass and lean body mass, cannot distinguish between lean body mass and fat mass, and provides no information on body fat distribution, it is necessary to check the correlation with HF using body composition rather than BMI (11).

Nevertheless, there have been few longitudinal studies targeting a general population of large sample sizes on the association of changes in body composition with the occurrence of HF. Therefore, we aimed to investigate the association between changes in body composition, including the predicted appendicular skeletal muscle mass index (pASMI), predicted body fat mass index (pBFMI), and predicted lean BMI (pLBMI), and the occurrence of HF in a longitudinal setting.

## 2 Materials and methods

### 2.1 Participants

**Figure 1:**
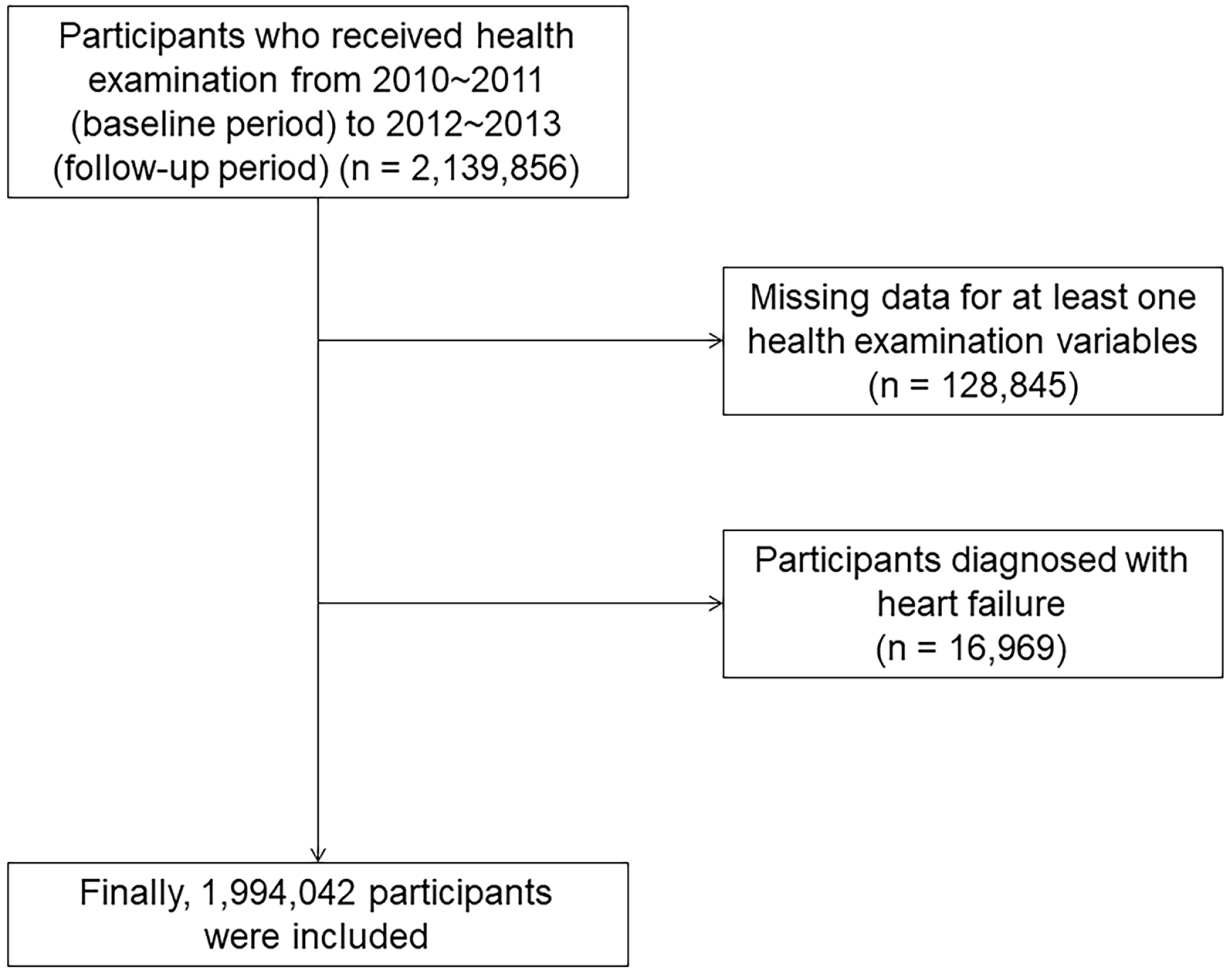
The flowchart of participant selection.

This study was performed using the National Health Insurance Service-Health Screening (NHIS-HEALS) dataset supplied by the Korean government. In South Korea, adults older than 40 years of age are advised to undergo free health examinations in alternate years. The Korean government combined the health screening dataset with age, sex, household income, and clinical information, including diagnostic codes, prescriptions, information on treatment or procedure, hospitalization, and mortality date (12, 13). For our study, 2,139,856 people who consecutively underwent national health examinations from 2010~2011 (baseline period) to 2012~2013 (follow-up period) were included (dataset number NHIS-2021-1-715) through an identification and validation process (14–16). Among 2,139,856 participants, those (n=85,947) with at least one missing data were excluded. Those with a previous history of HF (n=16,969) were excluded. Finally, 2,036,940 participants were included in the present study (**Figure 1**). The present study was approved by the Institutional Review Board of Ewha Womans University Seoul Hospital (Institutional Review Board approval number: SEUMC 2022-02-018).

### 2.2 Change in predicted body composition and covariates

The pBFMI, pASMI, and pLBMI were indirectly evaluated by validated anthropometric prediction equations from the Korean National Health and Nutrition Examination Survey cohort (11). A previous study suggested that body fat mass, appendicular skeletal muscle mass, and lean body mass were identified using dual-energy X-ray absorptiometry, and then a prediction equation was composed using different combinations of age, anthropometric measurements (weight, height, waist circumference), serum creatinine levels, physical activity, smoking status, and alcohol intake (11). These predictive equations were validated as having high predictive power, a moderate agreement rate, and low bias in the Korean general population (11). In the present study, these prediction equations were applied to evaluate the participants’ body composition. Body fat mass, appendicular skeletal muscle mass, and lean body mass were presented as indices (weight [kg] divided by height squared [m^2^]); thus, pBFMI, pASMI, and pLBMI, respectively (**Supplementary Methods 1**). The change in the pBFMI, termed ΔpBFMI, was calculated from the difference between the baseline and follow-up periods pBFMI, and the changes in the pASMI and pLBMI (ΔpASMI and ΔpLBMI) were also calculated similarly.

The detailed definitions of covariates were demonstrated in **Supplementary Methods 2** and previous studies (17–19). Variables including age, sex, BMI, household income (first, second, third, or fourth quartile), smoking status (never, former, or current), alcohol intake (0, 1-2, 3-4, or ≥5 times per week), physical activity (0, 1-2, 3-4, or ≥5 times per week), comorbid disease (hypertension, diabetes mellitus, dyslipidemia, atrial fibrillation, cancer, and renal disease), systolic blood pressure, fasting serum glucose, total cholesterol, estimated glomerular filtration rate, and Charlson Comorbidity Index (CCI) which is a commonly used method for evaluating comorbidities were collected (3, 18, 20). Comorbidities were defined considering the International Classification of Diseases, Tenth Revision (ICD-10) codes, history of prescriptions, and laboratory findings from the health examinations (3, 18).

### 2.3 Outcomes

The index date was defined as the date of the national health examination. The most recent health examination data were used for statistical analysis if more than one examination was performed during the period. Outcomes were defined as two or more claims for HF (21). Previously, the diagnostic accuracy of the ICD-10 code (I50) for HF using the NHIS dataset has been validated (3, 21). Monitoring was conducted until December 31, 2020, or the first occurrence of death or HF.

### 2.4 Statistical analysis

Chi-square and paired t-tests were used to compare categorical and continuous variables according to sex. The Cox proportional hazards model presented with a hazard ratio (HR) with a 95% confidence interval (CI) was used to evaluate the effect of changes in body composition (1 kg/m^2^ increments in the ΔpBFMI, ΔpASMI, and ΔpLBMI) between the baseline and follow-up periods on the occurrence of HF after adjusting for all potential confounding factors. In addition, we performed an additional multivariable analysis after adjusting for all potential confounding factors, except for variables used in the prediction equations to calculate the predicted body composition, including age, smoking status, alcohol intake, and physical activity. Furthermore, we provided restricted cubic splines of change in the predicted body composition to visually estimate the association between the change in the predicted body composition (ΔpBFMI, ΔpASMI, and ΔpLBMI) and the occurrence of HF. Four knots were placed at the 5th, 35th, 65th, and 95th percentiles of the change in the predicted body composition. Stratified analyses of the effects of changes in body composition on the occurrence of HF were conducted according to the subgroups of age, household income, alcohol intake, smoking status, physical activity, systolic blood pressure, fasting serum glucose, total cholesterol, CCI, and BMI at baseline and the follow-up categories: normal weight (18.5-24.9 kg/m^2^), overweight (25-29.9 kg/m^2^), and obese (≥ 30 kg/m^2^). To evaluate the combined effects of changes in the BMI, pASMI, pBFMI, and pLBMI, stratified analysis was additionally performed to evaluate the effect of changes in BMI during the two health examinations on the occurrence of HF according to changes in body composition. Statistical analyses were performed using R software, version 3.3.3 (R Foundation for Statistical Computing, Vienna, Austria) and SAS version 9.4 (SAS Inc., Cary, NC, USA). Two-sided *P*-values less than 0.05 were considered significant.

## 3 Results

**Table 1** shows the results of comparing the baseline characteristics of the 2,036,940 participants according to sex (n=1,070,377 for males, n=966,563 for females). During a median of 7.59 years of follow-up, 22,172 participants (event rate, 1.09%) with HF were observed. The mean age of males was 49.60 ± 13.45 years, and of females was 52.32 ± 13.65 years. Significant differences in the BMI at baseline and follow-up, household income, smoking status, alcohol intake, physical activity, systolic blood pressure, fasting serum glucose, total cholesterol, estimated glomerular filtration rate, and CCI were observed between males and females (**Table 1**). In the baseline period, the pASMI (23.67 ± 3.19 kg/m^2^ for males vs. 15.17 ± 1.85 kg/m^2^ for females), pBFMI (16.11 ± 4.27 kg/m^2^ for males vs. 19.16 ± 4.61 kg/m^2^ for females), and pLBMI (53.41 ± 6.52 kg/m^2^ for males vs. 37.25 ± 3.96 kg/m^2^ for females) were significantly different according to sex (*P*<0.001). In the follow-up period, the pASMI (23.68 ± 3.26 kg/m^2^ for males vs. 15.13 ± 1.86 kg/m^2^ for females), pBFMI (16.17 ± 4.34 kg/m^2^ for males vs. 19.22 ± 4.66 kg/m^2^ for females), and pLBMI (53.53 ± 6.66 kg/m^2^ for males vs. 37.23 ± 4.04 kg/m^2^ for females) were also significantly different according to sex (*P*<0.001, **Table 1**).

**Table 1:** Baseline characteristics of the study participants.

Variable	Total	Male	Female	*P*-value
Number of participants	2,036,940	1,070,377	966,563	<0.001
Age, years	50.89 ± 13.61	49.60 ± 13.45	52.32 ± 13.65	<0.001
Baseline period (2010–2011)				
BMI, kg/m^2^	23.79 ± 3.20	24.26 ± 3.03	23.28 ± 3.31	<0.001
pASMI, kg/m^2^	19.64 ± 5.00	23.67 ± 3.19	15.17 ± 1.85	<0.001
pBFMI, kg/m^2^	17.56 ± 4.69	16.11 ± 4.27	19.16 ± 4.61	<0.001
pLBMI, kg/m^2^	45.74 ± 9.74	53.41 ± 6.52	37.25 ± 3.96	<0.001
Follow-up period (2012–2013)				
BMI, kg/m^2^	23.86 ± 3.23	24.34 ± 3.07	23.33 ± 3.32	<0.001
pASMI, kg/m^2^	19.62 ± 5.04	23.68 ± 3.26	15.13 ± 1.86	<0.001
pBFMI, kg/m^2^	17.62 ± 4.74	16.17 ± 4.34	19.22 ± 4.66	<0.001
pLBMI, kg/m^2^	45.79 ± 9.86	53.53 ± 6.66	37.23 ± 4.04	<0.001
Household income				<0.001
First quintile, lowest	334,222 (16.41)	126,137 (11.78)	208,085 (21.53)	
Second quintile	390,984 (19.19)	180,343 (16.85)	210,641 (21.79)	
Third quintile	571,465 (28.06)	324,611 (30.33)	246,854 (25.54)	
Fourth quintile, highest	740,269 (36.34)	439,286 (41.04)	300,983 (31.14)	
Smoking status				<0.001
Never	1,256,017 (61.66)	329,914 (30.82)	926,103 (95.81)	
Former	336,000 (16.5)	321,040 (29.99)	14,960 (1.55)	
Current	444,923 (21.84)	419,423 (39.18)	25,500 (2.64)	
Alcohol intake, days/week				<0.001
<1	1,090,488 (53.54)	353,245 (33.00)	737,243 (76.27)	
1-2	674,668 (33.12)	480,738 (44.91)	193,930 (20.06)	
3-4	195,408 (9.59)	167,853 (15.68)	27,555 (2.85)	
≥5	76,376 (3.75)	68,541 (6.40)	7,835 (0.81)	
Physical activity, days/week				<0.001
<1	1,171,086 (57.49)	530,152 (49.53)	640,934 (66.31)	
1-2	514,062 (25.24)	332,208 (31.04)	181,854 (18.81)	
3-4	222,472 (10.92)	132,221 (12.35)	90,251 (9.34)	
≥5	129,320 (6.35)	75,796 (7.08)	53,524 (5.54)	
Systolic blood pressure, mmHg	122.29 ± 12.86	124.53 ± 11.78	119.81 ± 13.53	<0.001
Fasting serum glucose, mg/dL	98.21 ± 20.59	100.38 ± 22.29	95.8 ± 18.22	<0.001
Total cholesterol, mg/dL	195.75 ± 33.63	194.59 ± 33.45	197.04 ± 33.78	<0.001
Estimated glomerular filtration rate, mL/min/1.73m^2^				<0.001
<30	2,939 (0.14)	1,590 (0.15)	1,349 (0.14)	
30-60	115,170 (5.65)	54,748 (5.11)	60,422 (6.25)	
60-90	1,277,756 (62.73)	686,691 (64.15)	591,065 (61.15)	
≥90	641,075 (31.47)	327,348 (30.58)	313,727 (32.46)	
Charlson Comorbidity Index				<0.001
0	1,369,775 (67.25)	754,962 (70.53)	614,813 (63.61)	
1	446,919 (21.94)	214,619 (20.05)	232,300 (24.03)	
≥2	220,246 (10.81)	100,796 (9.42)	119,450 (12.36)	

P-values by Student’s t-test and chi-square test. Data are expressed as the mean ± SD deviation or n (%).

BMI, body mass index; pASMI, predicted appendicular skeletal muscle mass index; pBFMI, predicted body fat mass index; pLBMI, predicted lean body mass index.

**Figure 2:**
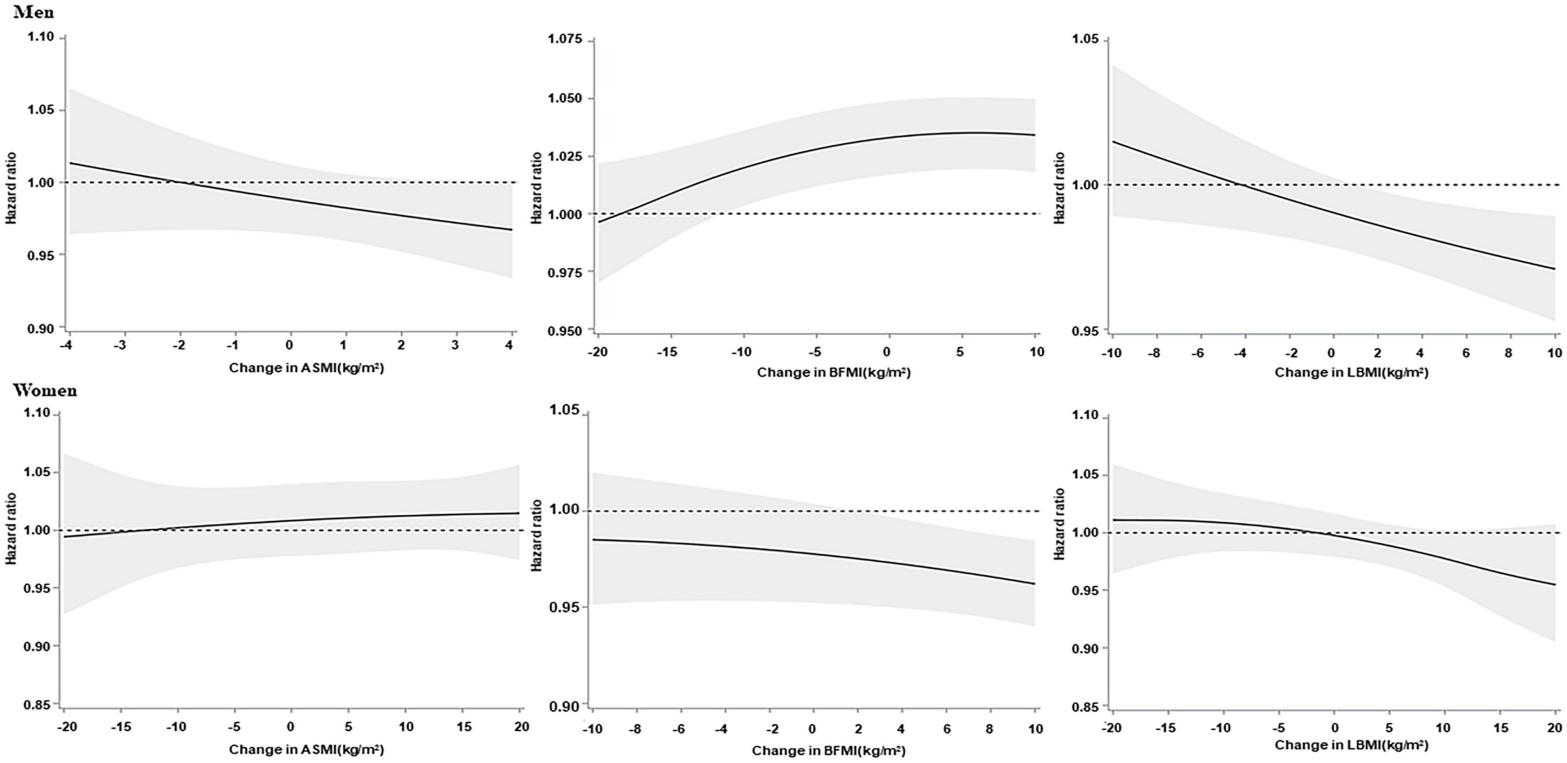
Association of the changes in the predicted body composition index with heart failure. Association of the changes in the predicted appendicular skeletal muscle mass index, body fat mass index, and lean body mass index (BMI) with heart failure. The solid lines indicate the hazard ratios, and the shaded regions show 95% confidence intervals from restricted cubic spline regression. Restricted cubic splines were constructed with four knots placed at the 5^th^, 35^th^, 65^th^, and 95^th^ percentiles of the change in the predicted appendicular skeletal muscle mass index, body fat mass index, and lean BMI. The hazard ratios (95% confidence interval) were calculated by Cox proportional hazards regression analysis after adjusting for age, BMI at baseline and follow-up, household income, smoking status, alcohol intake, physical activity, systolic blood pressure, fasting serum glucose, total cholesterol, and Charlson Comorbidity Index.

Multivariate analysis showed a relationship between the occurrence of HF and changes in body composition (**Table 2**; **Supplementary Table 1**). In males, with a 1 kg/m^2^ increase in the ΔpBFMI, the HR for the occurrence of HF was 1.017 (95%CI: 1.001-1.034), and the HRs for a 1 kg/m^2^ increase in the ΔpASMI and ΔpLBMI were 0.924 (95%CI: 0.900-0.947) and 0.951 (95%CI: 0.939-0.963), respectively. In females, with a 1 kg/m^2^ increase in the ΔpBFMI, the HR for the occurrence of HF was 0.925 (95%CI: 0.909-0.942), for a 1 kg/m^2^ increase in the ΔpASMI and ΔpLBMI were 0.966 (95%CI: 0.944-0.988), and 0.939 (95%CI: 0.923-0.955), respectively. In contrast, decreases in the ΔpASMI and ΔpLBMI were associated with an increased incidence of HF in both sexes. Furthermore, a decrease in the ΔpBFMI was associated with a decreased HF incidence in males, whereas a decrease in the ΔpBFMI was associated with an increased HF incidence in females (**Table 2**; **Supplementary Table 1** and **Figure 2**). For the stratified analysis, these trends and results were similar regardless of the BMI status at baseline. The occurrence-reducing effects of increased ΔpLBMI and ΔpASMI on the occurrence of HF were more prominent when the participants were less obese. Furthermore, the occurrence-reducing effect of an increased ΔpBFMI on the occurrence of HF was more prominent when female participants were less obese. In contrast to females, the occurrence-increasing effect of increased ΔpBFMI on the occurrence of HF was more prominent when male participants were less obese (**Table 3**; **Supplementary Table 2**).

**Table 2:** Hazard ratios and 95%CI of heart failure per 1 kg/m^2^ increase in the change in the predicted body composition index.

Variable	Male	*P*-value	Female	*P*-value
	HR (95%CI)		HR (95%CI)	
BMI at the baseline period, kg/m^2^	0.982 (0.961, 1.003)	0.096	1.043 (1.028, 1.059)	<0.001
BMI at the follow-up period, kg/m^2^	1.031 (1.009, 1.053)	0.006	1.015 (1.000, 1.031)	0.047
Household income				
First quintile, lowest	1(reference)		1(reference)	
Second quintile	0.723 (0.676, 0.772)	<0.001	0.876 (0.825, 0.929)	<0.001
Third quintile	0.677 (0.638, 0.719)	<0.001	0.978 (0.926, 1.033)	0.432
Fourth quintile, highest	0.773 (0.732, 0.817)	<0.001	1.190 (1.132, 1.250)	<0.001
Systolic blood pressure, mmHg	1.025 (1.023, 1.026)	<0.001	1.031 (1.029, 1.032)	<0.001
Fasting serum glucose, mg/dL	1.001 (1.000, 1.002)	0.021	1.002 (1.001, 1.003)	<0.001
Total cholesterol, mg/dL	0.994 (0.993, 0.994)	<0.001	0.997 (0.997, 0.998)	<0.001
Charlson Comorbidity Index				
0	1(reference)		1(reference)	
1	1.991 (1.902, 2.085)	<0.001	1.861 (1.782, 1.944)	<0.001
≥2	3.549 (3.376, 3.731)	<0.001	2.661 (2.538, 2.791)	<0.001
Change in the predicted body composition index				
ΔpASMI, kg/m^2^	0.924 (0.900, 0.947)	<0.001	0.966 (0.944, 0.988)	0.003
ΔpBFMI, kg/m^2^	1.017 (1.001, 1.034)	0.039	0.925 (0.909, 0.942)	<0.001
ΔpLBMI, kg/m^2^	0.951 (0.939, 0.963)	<0.001	0.939 (0.923, 0.955)	<0.001

The multivariable model was used for the BMI at the baseline and follow-up period, household income, systolic blood pressure, fasting serum glucose, total cholesterol, and Charlson Comorbidity Index.

BMI, body mass index; CI, confidence interval; HR, hazard ratio; ΔpASMI, change in predicted appendicular skeletal muscle mass index; ΔpBFMI, change in the predicted body fat mass index; ΔpLBMI, change in the predicted lean body mass index.

**Table 3:** Hazard ratios and 95%CI of heart failure per 1 kg/m^2^ increase in the change in the predicted body composition index stratified by the BMI group.

BMI at baseline period	Event	Person-years	ΔpASMI	*P*-value	pBFMI	*P*-value	ΔpLBMI	*P*-value
			HR (95%CI)		HR (95%CI)		HR (95%CI)	
Male								
Overall	10388	44622.87	0.924 (0.900, 0.947)	<0.001	1.017 (1.001, 1.034)	0.039	0.951 (0.939, 0.963)	<0.001
Normal weight	5784	25210.77	0.889 (0.857, 0.922)	<0.001	1.035 (1.011, 1.060)	0.003	0.928 (0.911, 0.946)	<0.001
Overweight	4144	17551.2	0.932 (0.896, 0.970)	<0.001	1.011 (0.985, 1.039)	0.403	0.959 (0.940, 0.978)	<0.001
Obese	460	1860.9	1.047 (0.998, 1.099)	0.061	0.991 (0.939, 1.047)	0.757	1.027 (0.999, 1.056)	0.055
Female								
Overall	11355	48062.99	0.966 (0.944, 0.988)	0.003	0.925 (0.909, 0.942)	<0.001	0.939 (0.923, 0.955)	<0.001
Normal weight	6273	26966.99	0.954 (0.928, 0.982)	0.001	0.919 (0.899, 0.939)	<0.001	0.919 (0.901, 0.938)	<0.001
Overweight	4253	17796.32	0.988 (0.946, 1.031)	0.581	0.936 (0.907, 0.967)	<0.001	0.959 (0.930, 0.988)	0.006
Obese	829	3299.68	0.992 (0.909, 1.083)	0.862	0.988 (0.922, 1.060)	0.744	1.004 (0.954, 1.056)	0.892

The multivariable model was used for the BMI at the baseline and follow-up periods, household income, systolic blood pressure, fasting serum glucose, total cholesterol, and Charlson Comorbidity Index.

BMI, body mass index; CI, confidence interval; HR, hazard ratio; ΔpASMI, change in the predicted appendicular skeletal muscle mass index; ΔpBFMI, change in the predicted body fat mass index; ΔpLBMI, change in the predicted lean body mass index.

**Table 4** shows the effect of BMI changes during the two health examinations on the occurrence of HF according to changes in body composition. Participants who maintained a stable weight (change in BMI ± 1 kg/m^2^) had a reduced incidence of HF occurrence per 1 kg/m^2^ increase in ΔpLBMI and ΔpASMI for males (HR:0.928, 95%CI 0.911-0.946; HR:0.894, 95%CI 0.863-0.927, respectively) and females (HR:0.923, 95%CI 0.904-0.943; HR:0.963, 95%CI 0.935-0.993, respectively). Male participants had an increased incidence of HF per 1 kg/m^2^ increase in ΔpBFMI (HR: 1.028; 95%CI: 1.005-1.050). However, female participants had a decreased incidence of HF per 1 kg/m^2^ increase in ΔpBFMI (HR: 0.918; 95%CI: 0.899-0.937; **Table 4**).

**Table 4:** Hazard ratios and 95%CI of heart failure per 1 kg/m^2^ increase in the change in the predicted body composition index stratified by a change in the BMI group.

Variables	Event	Person-years	ΔpASMI	*P*-value	ΔpBFMI	*P*-value	ΔpLBMI	*P*-value
			HR (95%CI)		HR (95%CI)		HR (95%CI)	
Male								
Stable weight	6995	30285.72	0.894 (0.863, 0.927)	<0.001	1.028 (1.005, 1.050)	0.014	0.928 (0.911, 0.946)	<0.001
Normal BMI at the baseline period								
Maintained normal	1346	5736.32	0.900 (0.837, 0.968)	0.004	1.041 (0.994, 1.091)	0.089	0.934 (0.898, 0.970)	<0.001
Normal to overweight	377	1628.47	0.904 (0.806, 1.013)	0.081	0.967 (0.893, 1.048)	0.411	0.945 (0.893, 1.001)	0.052
Normal to obese	7	30.44	0.970 (0.796, 1.181)	0.759	0.874 (0.675, 1.133)	0.309	0.986 (0.892, 1.089)	0.779
Overweight BMI at the baseline period								
Overweight to normal	383	1626.98	1.038 (0.925, 1.165)	0.524	1.102 (1.021, 1.19)	0.013	1.027 (0.967, 1.090)	0.382
Maintained overweight	851	3571.55	0.945 (0.863, 1.033)	0.213	0.970 (0.918, 1.024)	0.270	0.972 (0.927, 1.020)	0.248
Overweight to obese	107	440.71	0.843 (0.719, 0.988)	0.034	0.933 (0.830, 1.049)	0.247	0.917 (0.848, 0.992)	0.031
Obese BMI at the baseline period								
Obese to normal	7	34.98	1.128 (0.928, 1.371)	0.227	1.372 (0.990, 1.904)	0.057	1.067 (0.967, 1.178)	0.197
Obese to overweight	82	327.42	1.198 (0.990, 1.451)	0.063	1.044 (0.910, 1.197)	0.539	1.094 (0.999, 1.197)	0.052
Maintained obese	135	531.47	1.069 (0.930, 1.228)	0.347	0.986 (0.881, 1.104)	0.807	1.035 (0.966, 1.109)	0.332
Female								
Stable weight	6976	29686.87	0.963 (0.935, 0.993)	0.016	0.918 (0.899, 0.937)	<0.001	0.923 (0.904, 0.943)	<0.001
Normal BMI at the baseline period								
Maintained normal	1671	7168.64	0.960 (0.906, 1.017)	0.165	0.925 (0.876, 0.978)	<0.001	0.924 (0.887, 0.962)	<0.001
Normal to overweight	453	1895.6	0.931 (0.859, 1.008)	0.078	0.896 (0.867, 0.926)	<0.001	0.910 (0.856, 0.966)	0.002
Normal to obese	8	35.45	0.958 (0.697, 1.316)	0.790	0.911 (0.725, 1.145)	0.424	0.974 (0.84, 1.131)	0.731
Overweight BMI at the baseline period								
Overweight to normal	524	2241.71	1.041 (0.962, 1.128)	0.317	1.104 (0.987, 1.235)	0.082	1.036 (0.967, 1.110)	0.317
Maintained overweight	1036	4259.37	0.908 (0.859, 0.960)	<0.001	0.938 (0.866, 1.016)	0.118	0.901 (0.851, 0.953)	<0.001
Overweight to obese	138	583.26	1.065 (0.967, 1.173)	0.199	0.983 (0.861, 1.122)	0.795	1.056 (0.949, 1.174)	0.319
Obese BMI at the baseline period								
Obese to normal	8	24.51	0.934 (0.607, 1.439)	0.758	1.085 (0.832, 1.415)	0.548	0.978 (0.799, 1.197)	0.826
Obese to overweight	139	566.92	1.013 (0.874, 1.173)	0.867	1.038 (0.942, 1.145)	0.452	1.012 (0.939, 1.090)	0.757
Maintained obese	265	1069.9	1.022 (0.893, 1.169)	0.754	0.901 (0.823, 0.987)	0.025	0.998 (0.900, 1.107)	0.971

Multivariable model was adjusted for age, BMI at baseline and follow-up, household income, smoking status, alcohol intake, physical activity, systolic blood pressure, fasting serum glucose, total cholesterol, and Charlson Comorbidity Index. BMI, body mass index; CI, confidence interval; HR, hazard ratio; ΔpASMI, change in the predicted appendicular skeletal muscle mass index; ΔpBFMI, change in the predicted body fat mass index; ΔpLBMI, change in the predicted lean body mass index.

The occurrence-reducing effects of the increase in ΔpASMI and ΔpLBMI were observed in male participants whose BMI increased from normal to obese or overweight and from overweight to obese. The occurrence-reducing effect of the increase in the ΔpASMI and ΔpLBMI was also observed in female participants whose BMI increased from normal to overweight and obese. The decrease in the ΔpBFMI showed an occurrence-reducing effect in male participants whose BMI decreased from overweight or obese to normal and from obese to overweight. However, the increase in the ΔpBFMI showed an occurrence-reducing effect in female participants whose BMI increased from normal to obese or overweight and from overweight to obese (**Table 4**; **Supplementary Table 3**). In the subgroup analysis, there was no heterogeneity among the groups, except for age (**Supplementary Table 4**).

## 4 Discussion

In this large-scale cohort study, we found that decreased ΔpASMI and ΔpLBMI were negatively correlated with the occurrence of HF, regardless of sex. The occurrence-reducing effects of increased ΔpASMI and ΔpLBMI on the occurrence of HF were alleviated when the participants were more obese and when their BMI increased. In males, an increased ΔpBFMI showed a positive relationship with the occurrence of HF. The occurrence-increasing effect of increased ΔpBFMI on the occurrence of HF was more prominent when male participants were less obese and when the BMI of participants decreased. In contrast, decreased ΔpBFMI showed a negative relationship with the occurrence of HF in females. The occurrence-reducing effect of increased ΔpBFMI on the occurrence of HF was alleviated when the female participants were obese and when their BMI increased.

Previous studies showed that sarcopenia, which is primarily characterized by a progressive and widespread decline in skeletal muscle mass and muscle dysfunction, is common in patients with HF (about 10%) and is strongly related to the prognosis of HF (22). Also, loss of lean body mass was related to the occurrence of HF, particularly among older males (23). Various mechanisms, including malnutrition and anorexia, physical inactivity, and insulin resistance, cause an increase in muscle protein catabolism, resulting in a loss of muscle or body weight, which is associated with HF (24). Although present study could not confirm the causal relationship, in other hand, some studies suggested that muscle wasting is a consequence of HF and HF lead to several complications including musculoskeletal abnormalities and sarcopenia (25, 26). The present study showed that increased change in muscle mass had a negative relationship with the occurrence of HF, and the negative correlation was more attenuated when the participants were more obese and when the BMI of participants increased.

This study investigated sex-related differences in the association between changes in body fat mass and HF. Although the fat distribution varies according to sex, the role of obesity in the occurrence of HF in females may differ from that in males. Generally, males store sufficient fat in a visceral distribution, while females store sufficient fat in a peripheral subcutaneous distribution (27). These differences in location for stocking excessive fat have been shown to persist even after menopause. In males, visceral fat mass is associated with adiposopathy, which includes fat cell hypertrophy, increased circulating free fatty acids, and inflammatory and immune responses, leading to common metabolic diseases and HF (28, 29). The present study showed that an increased change in the body fat mass had a positive relationship with the occurrence of HF, and the occurrence-increasing effect of the increased change in body fat mass was more noticeable when the male participants were less obese and when the BMI of participants decreased. In females, peripherally distributed fat has a negative impact on exercise intolerance (30). Also, female patients with HF were found to be in a catabolic state and have more metabolic reserves. If patients experienced malnutrition, the burden of morbidity and mortality for HF increased, and loss of muscle mass occurred, leading to advanced HF (31). In contrast, obese patients showed low adiponectin levels and a decreased catecholamine response, which led to improved HF (32). Finally, obese females showed higher fatty acid uptake and less myocardial glucose utilization compared with obese males, which resulted in differences in myocardial metabolism (33, 34). The present study showed that increased changes in the body fat mass had a negative relationship with the occurrence of HF, and the occurrence-reducing effect of the increased change in body fat mass was more alleviated when the female participants were obese and when the BMI of participants increased.

The strength of our study is that it showed an association between changes in the body composition indices and the occurrence of HF in a large sample of the Korean general population in a longitudinal setting. However, our study has several limitations. First, present study has been conducted using anthropometric prediction equations to estimate body composition. Due to time constraint and cost, measurement of body composition using accurate modalities remains challenging issue in large epidemiological studies. Therefore, we decided to proceed with present study based on previous study which developed prediction equations to indirectly assess body composition using data obtained with dual-energy X-ray absorptiometry as reference (11). These anthropometric prediction equations from the Korean National Health and Nutrition Examination Survey cohort was validated, but it is major limitation of our study. Second, the equation for predicted body composition was estimated for the Korean population aged > 19 years; therefore, these findings may not be generalizable to other ethnicities and may not be directly applicable to different geographic and/or demographic settings. Third, although it was conducted with a longitudinal design, this is a retrospective study; therefore, we could not confirm the causal relationship or exclude confounders. Finally, because this is an epidemiologic study, our study cannot explain the basic mechanism of the association between changes in body composition and HF.

In conclusion, changes in body composition are associated with the occurrence of HF. Decreased ΔpASMI and ΔpLBMI are associated with the occurrence of HF. Furthermore, in males, increased ΔpBFMI was associated with the occurrence of HF. However, in females, decreased ΔpBFMI was associated with the occurrence of HF.

## Data availability statement

The raw data supporting the conclusions of this article will be made available by the authors, without undue reservation.

## Ethics statement

The studies involving humans were approved by Institutional Review Board approval number: SEUMC 2022-02-018. The studies were conducted in accordance with the local legislation and institutional requirements. Written informed consent for participation was not required from the participants or the participants’ legal guardians/next of kin in accordance with the national legislation and institutional requirements.

## Author contributions

T-JS had full access to all the data in the study and takes responsibility for the integrity of the data and the accuracy of the data analysis. HW, D-HK, HL, MKK, and T-JS conceived of and designed the study. T-JS and HL conducted the data acquisition and statistical analyses. HW, D-HK, MKK, and T-JS interpreted data and drafted the manuscript. T-JS reviewed and edited the manuscript. All authors contributed to the article and approved the submitted version.
