# Supplementary material for: Association between changes in predicted body composition and occurrence of heart failure: a nationwide population study
Source: Front Endocrinol (Lausanne). 2023 Oct 23;14:1210371. doi: 10.3389/fendo.2023.1210371 (PMC10627176; doi:10.3389/fendo.2023.1210371)
Supplement: Supplementary file 1 [file DataSheet_1.docx]

**Supplementary Table 1.** Hazard ratios and 95% confidence interval of heart failure per 1 kg/m^2^ increase in the change in the predicted body composition index

| Variable | Male |  | Female |  |
| --- | --- | --- | --- | --- |
|  | HR (95% CI) | *P*-value | HR (95% CI) | *P*-value |
| BMI at the baseline period, kg/m^2^ | 1.045 (1.024, 1.066) | 0.005 | 1.032 (1.019, 1.044) | <0.001 |
| BMI at the follow-up period, kg/m^2^ | 1.029 (1.010, 1.048) | 0.003 | 1.020 (1.007, 1.032) | 0.002 |
| Household income |  |  |  |  |
| First quintile, lowest | 1 (reference) |  | 1 (reference) |  |
| Second quintile | 0.824 (0.781, 0.869) | <0.001 | 0.926 (0.883, 0.972) | 0.002 |
| Third quintile | 0.756 (0.720, 0.794) | <0.001 | 0.874 (0.820, 0.924) | <0.001 |
| Fourth quintile, highest | 0.836 (0.800, 0.874) | <0.001 | 0.842 (0.803, 0.884) | <0.001 |
| Systolic blood pressure, mmHg | 1.023 (1.022, 1.024) | <0.001 | 1.028 (1.027, 1.029) | <0.001 |
| Fasting serum glucose, mg/dL | 1.000 (1.000, 1.001) | 0.374 | 1.000 (1.000, 1.001) | 0.182 |
| Total cholesterol, mg/dL | 0.998 (0.997, 0.998) | <0.001 | 1.000 (1.000, 1.000) | 0.986 |
| Comorbidities |  |  |  |  |
| Cancer | 1.006 (0.933, 1.085) | 0.880 | 1.005 (0.945, 1.064) | 0.973 |
| Renal disease | 1.703 (1.634, 1.775) | <0.001 | 1.525 (1.467, 1.585) | <0.001 |
| COPD | 1.638 (1.545, 1.737) | <0.001 | 1.734 (1.627, 1.848) | <0.001 |
| Liver disease | 1.081 (1.026, 1.137) | <0.001 | 1.052 (1.016, 1.087) | <0.001 |
| OSAS | 1.044 (1.023, 1.064) | 0.004 | 1.033 (0.999, 1.068) | 0.055 |
| Coronary artery disease | 1.108 (1.053, 1.164) | 0.003 | 1.064 (1.023, 1.106) | 0.007 |
| Cardiomyopathy | 2.477 (2.39, 2.568) | <0.001 | 2.201 (2.128, 2.276) | <0.001 |
| Valvular heart disease | 4.968 (4.333, 5.696) | <0.001 | 3.404 (2.864, 4.044) | <0.001 |
| Charlson Comorbidity Index |  |  |  |  |
| 0 | 1 (reference) |  | 1 (reference) |  |
| 1 | 1.645 (1.571, 1.721) | <0.001 | 1.690 (1.606, 1.778) | <0.001 |
| ≥2 | 2.550 (2.436, 2.669) | <0.001 | 2.698 (2.571, 2.831) | <0.001 |
| Change in the predicted body composition index |  |  |  |  |
| ΔpASMI, kg/m^2^ | 0.952 (0.932, 0.972) | <0.001 | 0.960 (0.940, 0.979) | <0.001 |
| ΔpBFMI, kg/m^2^ | 1.023 (1.001, 1.044) | 0.028 | 0.913 (0.894, 0.933) | <0.001 |
| ΔpLBMI, kg/m^2^ | 0.967 (0.956, 0.977) | <0.001 | 0.938 (0.924, 0.953) | <0.001 |

The multivariable model was used for the BMI at the baseline and follow-up period, household income, systolic blood pressure, fasting serum glucose, total cholesterol, cancer, renal disease, COPD, liver disease, OSAS, coronary artery disease, cardiomyopathy, valvular heart disease, and Charlson Comorbidity Index.

BMI, body mass index; CI, confidence interval; COPD: Chronic obstructive pulmonary disease; HR, hazard ratio; OSAS: Obstructive sleep apnea syndrome; ΔpASMI, change in predicted appendicular skeletal muscle mass index; ΔpBFMI, change in the predicted body fat mass index; ΔpLBMI, change in the predicted lean body mass index.

**Supplementary Table 2.** Hazard ratios and 95% confidence interval of heart failure per 1 kg/m^2^ increase in change in predicted body composition index stratified by the body mass index group

|  |  |  | ΔpASMI |  | ΔpBFMI |  | ΔpLBMI |  |
| --- | --- | --- | --- | --- | --- | --- | --- | --- |
| BMI at baseline period | Event | Person-years | HR (95% CI) | *P* | HR (95% CI) | *P* | HR (95% CI) | *P* |
| Male |  |  |  |  |  |  |  |  |
| Overall | 10388 | 44622.87 | 0.984 (0.961, 1.008) | 0.183 | 1.026 (1.009, 1.042) | 0.001 | 0.988 (0.976, 1.000) | 0.049 |
| Normal weight | 5784 | 25210.77 | 0.962 (0.929, 0.997) | 0.031 | 1.046 (1.022, 1.070) | <.001 | 0.974 (0.956, 0.992) | 0.005 |
| Overweight | 4144 | 17551.2 | 0.984 (0.949, 1.021) | 0.397 | 1.017 (0.991, 1.043) | 0.194 | 0.990 (0.972, 1.008) | 0.267 |
| Obese | 460 | 1860.9 | 1.053 (0.997, 1.112) | 0.065 | 0.976 (0.931, 1.024) | 0.318 | 1.029 (0.997, 1.061) | 0.076 |
| Female |  |  |  |  |  |  |  |  |
| Overall | 11355 | 48062.99 | 1.009 (0.984, 1.034) | 0.485 | 0.969 (0.946, 0.994) | 0.013 | 0.995 (0.977, 1.013) | 0.575 |
| Normal weight | 6273 | 26966.99 | 1.002 (0.967, 1.039) | 0.909 | 0.970 (0.935, 1.006) | 0.105 | 0.985 (0.960, 1.010) | 0.229 |
| Overweight | 4253 | 17796.32 | 1.019 (0.981, 1.057) | 0.329 | 0.958 (0.923, 0.994) | 0.023 | 1.001 (0.971, 1.032) | 0.933 |
| Obese | 829 | 3299.68 | 1.008 (0.927, 1.096) | 0.857 | 0.993 (0.928, 1.062) | 0.833 | 1.014 (0.968, 1.062) | 0.558 |

Multivariable model was adjusted for age, body mass index at baseline and follow-up period, household income, smoking status, alcohol intake, physical activity, systolic blood pressure, fasting serum glucose, total cholesterol, Charlson Comorbidity Index

BMI, body mass index, CI, confidence interval; HR, hazard ratio; ΔpASMI, change in predicted appendicular skeletal muscle mass index; ΔpBFMI, change in predicted body fat mass index; ΔpLBMI, change in predicted lean body mass index.

**Supplementary Table 3.** Hazard ratios and 95% confidence interval of heart failure per 1 kg/m^2^ increase in change in predicted body composition index stratified by change in body mass index group

| Variables | Event | Person-years | ΔpASMI |  | ΔpBFMI |  | ΔpLBMI |  |
| --- | --- | --- | --- | --- | --- | --- | --- | --- |
|  |  |  | HR (95% CI) | *P* | HR (95% CI) | *P* | HR (95% CI) | *P* |
| Male |  |  |  |  |  |  |  |  |
| Stable weight | 6995 | 30285.72 | 0.958 (0.925, 0.992) | 0.016 | 1.027 (1.005, 1.049) | 0.015 | 0.969 (0.950, 0.987) | <.001 |
| Normal BMI at baseline period |  |  |  |  |  |  |  |  |
| Maintained normal | 1346 | 5736.32 | 0.967 (0.902, 1.036) | 0.339 | 1.050 (1.003, 1.100) | 0.037 | 0.973 (0.937, 1.010) | 0.150 |
| Normal to overweight | 377 | 1628.47 | 1.020 (0.916, 1.136) | 0.718 | 1.073 (0.994, 1.159) | 0.072 | 1.017 (0.961, 1.075) | 0.566 |
| Normal to obese | 7 | 30.44 | 1.036 (0.818, 1.313) | 0.767 | 0.884 (0.628, 1.244) | 0.478 | 1.021 (0.904, 1.153) | 0.741 |
| Overweight BMI at baseline period |  |  |  |  |  |  |  |  |
| Overweight to normal | 383 | 1626.98 | 1.009 (0.904, 1.126) | 0.877 | 1.040 (0.960, 1.126) | 0.336 | 1.002 (0.946, 1.062) | 0.933 |
| Maintained overweight | 851 | 3571.55 | 1.000 (0.921, 1.087) | 0.994 | 0.985 (0.938, 1.035) | 0.547 | 1.004 (0.960, 1.049) | 0.862 |
| Overweight to obese | 107 | 440.71 | 0.930 (0.801, 1.081) | 0.344 | 1.055 (0.931, 1.196) | 0.402 | 0.967 (0.896, 1.043) | 0.386 |
| Obese BMI at baseline period |  |  |  |  |  |  |  |  |
| Obese to normal | 7 | 34.98 | 1.025 (0.760, 1.383) | 0.872 | 3.630 (0.746, 17.671) | 0.110 | 1.02 (0.875, 1.189) | 0.796 |
| Obese to overweight | 82 | 327.42 | 1.137 (0.943, 1.371) | 0.179 | 0.987 (0.858, 1.136) | 0.855 | 1.061 (0.968, 1.162) | 0.207 |
| Maintained obese | 135 | 531.47 | 1.096 (0.955, 1.258) | 0.193 | 0.990 (0.891, 1.101) | 0.854 | 1.049 (0.980, 1.123) | 0.169 |
| Female |  |  |  |  |  |  |  |  |
| Stable weight | 6976 | 29686.87 | 1.006 (0.970, 1.043) | 0.745 | 0.946 (0.917, 0.975) | <.001 | 0.982 (0.954, 1.010) | 0.209 |
| Normal BMI at baseline period |  |  |  |  |  |  |  |  |
| Maintained normal | 1671 | 7168.64 | 1.006 (0.947, 1.069) | 0.850 | 0.994 (0.926, 1.068) | 0.877 | 0.986 (0.942, 1.031) | 0.532 |
| Normal to overweight | 453 | 1895.6 | 1.032 (0.941, 1.132) | 0.499 | 0.929 (0.856, 1.008) | 0.078 | 1.013 (0.946, 1.084) | 0.713 |
| Normal to obese | 8 | 35.45 | 1.075 (0.751, 1.539) | 0.693 | 1.039 (0.780, 1.385) | 0.793 | 1.027 (0.868, 1.216) | 0.753 |
| Overweight BMI at baseline period |  |  |  |  |  |  |  |  |
| Overweight to normal | 524 | 2241.71 | 1.025 (0.928, 1.132) | 0.625 | 0.929 (0.837, 1.031) | 0.166 | 1.008 (0.940, 1.081) | 0.829 |
| Maintained overweight | 1036 | 4259.37 | 0.940 (0.869, 1.017) | 0.125 | 1.000 (0.914, 1.094) | 0.995 | 0.964 (0.906, 1.026) | 0.244 |
| Overweight to obese | 138 | 583.26 | 1.100 (1.020, 1.187) | 0.013 | 1.224 (1.003, 1.495) | 0.047 | 1.125 (1.034, 1.224) | 0.006 |
| Obese BMI at baseline period |  |  |  |  |  |  |  |  |
| Obese to normal | 8 | 24.51 | 0.872 (0.565, 1.346) | 0.536 | 0.984 (0.697, 1.388) | 0.926 | 0.938 (0.755, 1.166) | 0.5653 |
| Obese to overweight | 139 | 566.92 | 0.986 (0.868, 1.121) | 0.831 | 0.967 (0.874, 1.069) | 0.509 | 0.990 (0.923, 1.063) | 0.788 |
| Maintained obese | 265 | 1069.9 | 1.048 (0.964, 1.140) | 0.267 | 0.974 (0.855, 1.109) | 0.687 | 1.051 (0.968, 1.142) | 0.232 |

Multivariable model was adjusted for age, body mass index at baseline and follow-up period, household income, smoking status, alcohol intake, physical activity, systolic blood pressure, fasting serum glucose, total cholesterol, Charlson Comorbidity Index

BMI, body mass index; CI, confidence interval; HR, hazard ratio; ΔpASMI, change in predicted appendicular skeletal muscle mass index; ΔpBFMI, change in predicted body fat mass index; ΔpLBMI, change in predicted lean body mass index.

**Supplementary Table 4.** Subgroup analysis of the association of changes in predicted body composition index with heart failure

| Variable |  |  | Male |  |  |  |  |  | Female |  |  |  |
| --- | --- | --- | --- | --- | --- | --- | --- | --- | --- | --- | --- | --- |
|  | ΔpASMI |  | ΔpBFMI |  | ΔpLBMI |  | ΔpASMI |  | ΔpBFMI |  | ΔpLBMI |  |
|  | HR (95% CI) | *P* | HR (95% CI) | *P* | HR (95% CI) | *P* | HR (95% CI) | *P* | HR (95% CI) | *P* | HR (95% CI) | *P* |
| Age, years |  | 0.906 |  | 0.0007 |  | 0.941 |  | 0.006 |  | 0.006 |  | 0.001 |
| <65 | 0.981 (0.966, 0.997) |  | 0.995 (0.987, 1.003) |  | 0.987 (0.980, 0.995) |  | 0.983 (0.964, 1.002) |  | 0.999 (0.985, 1.014) |  | 0.987 (0.976, 0.997) |  |
| ≥65 | 0.982 (0.956, 1.008) |  | 1.016 (0.999, 1.034) |  | 0.991 (0.977, 1.005) |  | 0.973 (0.945, 1.002) |  | 0.968 (0.940, 0.996) |  | 0.972 (0.948, 0.997) |  |
| BMI at baseline period, kg/m^2^ |  | 0.440 |  | 0.126 |  | 0.376 |  | 0.905 |  | 0.327 |  | 0.933 |
| <25 | 0.967 (0.935, 1.001) |  | 1.041 (1.018, 1.064) |  | 0.976 (0.959, 0.994) |  | 1.002 (0.968, 1.038) |  | 0.973 (0.938, 1.009) |  | 0.986 (0.962, 1.011) |  |
| ≥25 | 0.997 (0.964, 1.030) |  | 1.007 (0.985, 1.030) |  | 0.996 (0.980, 1.013) |  | 1.015 (0.981, 1.050) |  | 0.965 (0.933, 0.997) |  | 1.003 (0.979, 1.028) |  |
| BMI at follow-up period, kg/m^2^ |  | 0.427 |  | 0.234 |  | 0.289 |  | 0.167 |  | 0.771 |  | 0.075 |
| <25 | 0.966 (0.933, 1.001) |  | 1.037 (1.014, 1.060) |  | 0.973 (0.955, 0.992) |  | 0.990 (0.956, 1.025) |  | 0.966 (0.932, 1.001) |  | 0.977 (0.954, 1.002) |  |
| ≥25 | 0.999 (0.967, 1.033) |  | 1.012 (0.989, 1.035) |  | 0.999 (0.982, 1.016) |  | 1.027 (0.995, 1.060) |  | 0.971 (0.939, 1.004) |  | 1.013 (0.988, 1.039) |  |
| Household income |  | 0.361 |  | 0.263 |  | 0.353 |  | 0.378 |  | 0.770 |  | 0.844 |
| First quartile, lowest | 1.030 (0.971, 1.093) |  | 1.026 (0.987, 1.067) |  | 1.004 (0.973, 1.036) |  | 1.058 (1.014, 1.104) |  | 0.940 (0.886, 0.998) |  | 1.034 (0.994, 1.075) |  |
| Second quartile | 0.973 (0.917, 1.032) |  | 1.060 (1.019, 1.104) |  | 0.990 (0.959, 1.021) |  | 0.982 (0.917, 1.052) |  | 0.965 (0.907, 1.027) |  | 0.987 (0.944, 1.032) |  |
| Third quartile | 0.990 (0.948, 1.033) |  | 1.025 (0.994, 1.057) |  | 0.996 (0.974, 1.018) |  | 1.028 (0.978, 1.080) |  | 0.964 (0.920, 1.011) |  | 0.996 (0.959, 1.035) |  |
| Fourth quartile, highest | 0.964 (0.927, 1.003) |  | 1.012 (0.987, 1.038) |  | 0.973 (0.954, 0.994) |  | 0.981 (0.942, 1.022) |  | 0.987 (0.949, 1.027) |  | 0.980 (0.953, 1.007) |  |
| Smoking status |  | 0.102 |  | 0.240 |  | 0.108 |  | 0.532 |  | 0.823 |  | 0.695 |
| Never | 0.997 (0.958, 1.038) |  | 1.024 (0.997, 1.051) |  | 0.990 (0.969, 1.011) |  | 1.006 (0.981, 1.032) |  | 0.971 (0.947, 0.995) |  | 0.993 (0.975, 1.011) |  |
| Former | 0.979 (0.941, 1.019) |  | 1.027 (1.001, 1.055) |  | 0.989 (0.968, 1.009) |  | 1.112 (0.821, 1.505) |  | 0.940 (0.774, 1.142) |  | 1.025 (0.866, 1.212) |  |
| Current | 0.973 (0.930, 1.017) |  | 1.026 (0.994, 1.060) |  | 0.983 (0.961, 1.006) |  | 1.065 (0.957, 1.184) |  | 0.924 (0.790, 1.080) |  | 1.052 (0.938, 1.178) |  |
| Alcohol intake, days/week |  | 0.859 |  | 0.327 |  | 0.887 |  | 0.979 |  | 0.857 |  | 0.988 |
| <1 | 0.994 (0.961, 1.028) |  | 1.044 (1.020, 1.068) |  | 0.992 (0.975, 1.010) |  | 1.012 (0.986, 1.038) |  | 0.967 (0.942, 0.992) |  | 0.996 (0.978, 1.015) |  |
| 1-2 | 0.966 (0.925, 1.010) |  | 1.005 (0.976, 1.035) |  | 0.983 (0.961, 1.006) |  | 0.996 (0.913, 1.086) |  | 1.017 (0.932, 1.110) |  | 1.006 (0.944, 1.072) |  |
| 3-4 | 0.942 (0.877, 1.012) |  | 1.025 (0.979, 1.073) |  | 0.953 (0.918, 0.990) |  | 0.930 (0.767, 1.128) |  | 0.891 (0.759, 1.045) |  | 0.892 (0.782, 1.018) |  |
| ≥5 | 1.031 (0.955, 1.112) |  | 0.988 (0.937, 1.042) |  | 1.014 (0.974, 1.055) |  | 0.948 (0.832, 1.080) |  | 0.859 (0.587, 1.257) |  | 0.912 (0.820, 1.013) |  |
| Physical activity, days/week |  | 0.207 |  | 0.185 |  | 0.240 |  | 0.713 |  | 0.572 |  | 0.456 |
| <1 | 0.986 (0.956, 1.018) |  | 1.023 (1.002, 1.045) |  | 0.989 (0.972, 1.005) |  | 1.010 (0.982, 1.039) |  | 0.966 (0.94, 0.992) |  | 0.995 (0.975, 1.014) |  |
| 1-2 | 0.973 (0.928, 1.020) |  | 1.046 (1.012, 1.082) |  | 0.985 (0.962, 1.008) |  | 0.989 (0.911, 1.073) |  | 0.992 (0.914, 1.077) |  | 0.980 (0.925, 1.038) |  |
| 3-4 | 0.993 (0.927, 1.064) |  | 1.028 (0.980, 1.079) |  | 0.992 (0.959, 1.027) |  | 1.023 (0.931, 1.123) |  | 1.046 (0.940, 1.163) |  | 1.027 (0.953, 1.106) |  |
| ≥5 | 1.014 (0.932, 1.103) |  | 0.991 (0.939, 1.045) |  | 1.000 (0.957, 1.044) |  | 1.004 (0.901, 1.120) |  | 0.925 (0.852, 1.004) |  | 0.991 (0.908, 1.081) |  |
| Systolic blood pressure, mmHg |  | 0.526 |  | 0.550 |  | 0.799 |  | 0.593 |  | 0.171 |  | 0.317 |
| <130 | 0.973 (0.941, 1.006) |  | 1.027 (1.004, 1.050) |  | 0.984 (0.967, 1.001) |  | 1.013 (0.980, 1.047) |  | 0.954 (0.921, 0.987) |  | 0.996 (0.972, 1.021) |  |
| ≥130 | 0.994 (0.960, 1.030) |  | 1.023 (0.999, 1.047) |  | 0.990 (0.973, 1.009) |  | 1.005 (0.968, 1.043) |  | 0.985 (0.949, 1.022) |  | 0.994 (0.968, 1.020) |  |
| Fasting serum glucose, mg/dL |  | 0.7378 |  | 0.722 |  | 0.725 |  | 0.841 |  | 0.190 |  | 0.483 |
| <126 | 0.988 (0.963, 1.013) |  | 1.024 (1.007, 1.042) |  | 0.990 (0.977, 1.003) |  | 1.013 (0.987, 1.040) |  | 0.971 (0.946, 0.998) |  | 0.998 (0.979, 1.017) |  |
| ≥126 | 0.960 (0.898, 1.027) |  | 1.035 (0.987, 1.085) |  | 0.973 (0.941, 1.007) |  | 0.973 (0.890, 1.063) |  | 0.955 (0.902, 1.012) |  | 0.972 (0.919, 1.028) |  |
| Total cholesterol, mg/dL |  | 0.257 |  | 0.008 |  | 0.177 |  | 0.171 |  | 0.452 |  | 0.499 |
| <200 | 0.977 (0.949, 1.006) |  | 1.033 (1.013, 1.053) |  | 0.984 (0.969, 0.999) |  | 1.028 (0.998, 1.060) |  | 0.957 (0.926, 0.988) |  | 1.006 (0.982, 1.030) |  |
| ≥200 | 0.996 (0.956, 1.038) |  | 1.011 (0.983, 1.039) |  | 0.994 (0.973, 1.016) |  | 0.981 (0.943, 1.021) |  | 0.985 (0.949, 1.023) |  | 0.982 (0.956, 1.008) |  |
| Charlson comorbidity index |  | 0.878 |  | 0.345 |  | 0.664 |  | 0.951 |  | 0.963 |  | 0.900 |
| 0 | 0.987 (0.951, 1.024) |  | 1.007 (0.983, 1.031) |  | 0.992 (0.973, 1.011) |  | 1.017 (0.978, 1.057) |  | 0.974 (0.932, 1.016) |  | 1.005 (0.976, 1.036) |  |
| 1 | 0.984 (0.943, 1.027) |  | 1.039 (1.009, 1.071) |  | 0.989 (0.968, 1.011) |  | 1.000 (0.953, 1.049) |  | 0.960 (0.918, 1.004) |  | 0.984 (0.953, 1.015) |  |
| ≥2 | 0.976 (0.930, 1.023) |  | 1.041 (1.010, 1.074) |  | 0.978 (0.954, 1.002) |  | 1.008 (0.967, 1.052) |  | 0.972 (0.933, 1.012) |  | 0.995 (0.965, 1.027) |  |

Multivariable model was for body mass index at baseline and follow-up period, household income, systolic blood pressure, fasting serum glucose, total cholesterol, Charlson Comorbidity Index

BMI, body mass index; CI, confidence interval; HR, hazard ratio; ΔpASMI, change in predicted appendicular skeletal muscle mass index; ΔpBFMI, change in predicted body fat mass index; ΔpLBMI, change in predicted lean body mass index.

**Supplementary Table 6.** baseline characteristics of study participants: age 65 and older vs. under age 65

| Variable | Total | Age<65 | Age≥65 | P-value |
| --- | --- | --- | --- | --- |
| Number of participants | 1,994,042 | 1,657,163 | 336,879 | <0.001 |
| Age, years | 50.77±13.53 | 46.54±10.47 | 71.58±4.90 | <0.001 |
| Baseline period (2010-2011) |  |  |  |  |
| BMI, kg/m^2^ | 23.79±3.20 | 23.74±3.23 | 24.06±3.03 | <0.001 |
| pASMI, kg/m^2^ | 19.67±5.00 | 20.09±5.07 | 17.61±4.02 | <0.001 |
| pBFMI, kg/m^2^ | 17.54±4.68 | 17.56±4.66 | 17.47±4.78 | <0.001 |
| pLBMI, kg/m^2^ | 45.79±9.75 | 46.51±9.89 | 42.22±8.09 | <0.001 |
| Follow-up period (2012-2013) |  |  |  |  |
| BMI, kg/m^2^ | 23.86±3.23 | 23.84±3.26 | 23.94±3.07 | <0.001 |
| pASMI, kg/m^2^ | 19.65±5.04 | 20.10±5.11 | 17.44±4.02 | <0.001 |
| pBFMI, kg/m^2^ | 17.61±4.74 | 17.68±4.72 | 17.25±4.80 | <0.001 |
| pLBMI, kg/m^2^ | 45.85±9.87 | 46.64±10.00 | 41.92±8.12 | <0.001 |
| Household income |  |  |  | <0.001 |
| First quintile, lowest | 330,822 (16.59) | 266,657 (16.09) | 64,165 (19.05) |  |
| Second quintile | 391,555 (19.64) | 343,280 (20.71) | 48,275 (14.33) |  |
| Third quintile | 555,574 (27.86) | 479,275 (28.92) | 76,299 (22.65) |  |
| Fourth quintile, highest | 716,091 (35.91) | 567,951 (34.27) | 148,140 (43.97) |  |
| Smoking status |  |  |  | <0.001 |
| Never | 1,224,927 (61.43) | 983,095 (59.32) | 241,832 (71.79) |  |
| Former | 329,560 (16.53) | 269,201 (16.24) | 60,359 (17.92) |  |
| Current | 439,555 (22.04) | 404,867 (24.43) | 34,688 (10.30) |  |
| Alcohol intake, days/week |  |  |  | <0.001 |
| <1 | 1,059,925 (53.15) | 806,038 (48.64) | 253,887 (75.36) |  |
| 1-2 | 665,061 (33.35) | 619,650 (37.39) | 45,411 (13.48) |  |
| 3-4 | 192,957 (9.68) | 174,031 (10.50) | 18,926 (5.62) |  |
| ≥5 | 76,099 (3.82) | 57,444 (3.47) | 18,655 (5.54) |  |
| Physical activity, days/week |  |  |  | <0.001 |
| <1 | 1,141,836 (57.26) | 903,007 (54.49) | 238,829 (70.89) |  |
| 1-2 | 506,003 (25.38) | 464,707 (28.04) | 41,296 (12.26) |  |
| 3-4 | 218,614 (10.96) | 190,515 (11.5) | 28,099 (8.34) |  |
| ≥5 | 127,589 (6.40) | 98,934 (5.97) | 28,655 (8.51) |  |
| Systolic blood pressure, mmHg | 122.27±12.84 | 120.88±12.41 | 129.12±12.72 | <0.001 |
| Fasting serum glucose, mg/dL | 98.15±20.53 | 97.06±19.95 | 103.52±22.42 | <0.001 |
| Total cholesterol, mg/dL | 195.91±33.6 | 196.11±33.3 | 194.92±34.99 | <0.001 |
| Estimated glomerular filtration rate, mL/min/1.73m^2^ |  |  |  | <0.001 |
| <30 | 2,603 (0.13) | 1,381 (0.08) | 1,222 (0.36) |  |
| 30-60 | 110,882 (5.56) | 62,574 (3.78) | 48,308 (14.34) |  |
| 60-90 | 1,252,703 (62.82) | 1,029,937 (62.15) | 222,766 (66.13) |  |
| ≥90 | 627,854 (31.49) | 563,271 (33.99) | 64,583 (19.17) |  |
| Comorbidities |  |  |  |  |
| Hypertension | 650,280 (32.61) | 421,795 (25.45) | 228,485 (67.82) | <0.001 |
| Diabetes mellitus | 315,533 (15.82) | 200,093 (12.07) | 115,440 (34.27) | <0.001 |
| Dyslipidemia | 688,439 (34.52) | 504,377 (30.44) | 184,062 (54.64) | <0.001 |
| Cancer | 52,166 (2.62) | 34,275 (2.07) | 17,891 (5.31) | <0.001 |
| Renal disease | 153,551 (7.7) | 90,024 (5.43) | 63,527 (18.86) | <0.001 |
| COPD | 40,069 (2.01) | 16,906 (1.02) | 23,163 (6.88) | <0.001 |
| Liver disease | 404,570 (20.29) | 305,549 (18.44) | 99,021 (29.39) | <0.001 |
| OSAS | 1,934 (0.1) | 1,758 (0.11) | 176 (0.05) | <0.001 |
| Coronary artery disease | 219,096 (10.99) | 131,523 (7.94) | 87,573 (26) | <0.001 |
| Cardiomyopathy | 1,854 (0.09) | 1,045 (0.06) | 809 (0.24) | <0.001 |
| Valvular heart disease | 6,352 (0.32) | 3,336 (0.2) | 3,016 (0.9) | <0.001 |
| Charlson Comorbidity Index |  |  |  | <0.001 |
| 0 | 856,970 (42.98) | 799,324 (48.23) | 57,646 (17.11) |  |
| 1 | 563,577 (28.26) | 480,913 (29.02) | 82,664 (24.54) |  |
| ≥2 | 573,495 (28.76) | 376,926 (22.75) | 196,569 (58.35) |  |

P-values by Student’s t-test and chi-square test. Data are expressed as the mean±SD deviation or n (%).

BMI, body mass index; COPD, chronic obstructive pulmonary disease; OSAS, obstructive sleep apnea syndrome; pASMI, predicted appendicular skeletal muscle mass index; pBFMI, predicted body fat mass index; pLBMI, predicted lean body mass index. **Supplementary Table 7.** Baseline characteristics of the study participants aged 65 and older, grouped by gender

| Variable | Total | Men | Women | *P*-value |
| --- | --- | --- | --- | --- |
| Number of participants | 336,879 | 158,658 | 178,221 | <0.001 |
| Age, years | 71.58±4.90 | 71.5±4.87 | 71.64±4.91 | <0.001 |
| Baseline period (2010-2011) |  |  |  |  |
| BMI, kg/m^2^ | 24.06±3.03 | 23.72±2.85 | 24.35±3.16 | <0.001 |
| pASMI, kg/m^2^ | 17.61±4.02 | 21.16±2.58 | 14.45±1.86 | <0.001 |
| pBFMI, kg/m^2^ | 17.47±4.78 | 15.05±3.94 | 19.63±4.41 | <0.001 |
| pLBMI, kg/m^2^ | 42.22±8.09 | 49.15±5.45 | 36.05±4.04 | <0.001 |
| Follow-up period (2012-2013) |  |  |  |  |
| BMI, kg/m^2^ | 23.94±3.07 | 23.61±2.88 | 24.23±3.21 | <0.001 |
| pASMI, kg/m^2^ | 17.44±4.02 | 20.98±2.60 | 14.28±1.87 | <0.001 |
| pBFMI, kg/m^2^ | 17.25±4.80 | 14.87±3.98 | 19.37±4.47 | <0.001 |
| pLBMI, kg/m^2^ | 41.92±8.12 | 48.86±5.49 | 35.75±4.09 | <0.001 |
| Household income |  |  |  | <0.001 |
| First quintile, lowest | 64,165 (19.05) | 31,605 (19.92) | 32,560 (18.27) |  |
| Second quintile | 48,275 (14.33) | 24,029 (15.15) | 24,246 (13.6) |  |
| Third quintile | 76,299 (22.65) | 36,543 (23.03) | 39,756 (22.31) |  |
| Fourth quintile, highest | 148,140 (43.97) | 66,481 (41.9) | 81,659 (45.82) |  |
| Smoking status |  |  |  | <0.001 |
| Never | 241,832 (71.79) | 67,579 (42.59) | 174,253 (97.77) |  |
| Former | 60,359 (17.92) | 58,973 (37.17) | 1,386 (0.78) |  |
| Current | 34,688 (10.30) | 32,106 (20.24) | 2,582 (1.45) |  |
| Alcohol intake, days/week |  |  |  | <0.001 |
| <1 | 253,887 (75.36) | 86,003 (54.21) | 167,884 (94.2) |  |
| 1-2 | 45,411 (13.48) | 37,395 (23.57) | 8,016 (4.5) |  |
| 3-4 | 18,926 (5.62) | 17,649 (11.12) | 1,277 (0.72) |  |
| ≥5 | 18,655 (5.54) | 17,611 (11.1) | 1,044 (0.59) |  |
| Physical activity, days/week |  |  |  | <0.001 |
| <1 | 238,829 (70.89) | 100,637 (63.43) | 138,192 (77.54) |  |
| 1-2 | 41,296 (12.26) | 23,512 (14.82) | 17,784 (9.98) |  |
| 3-4 | 28,099 (8.34) | 16,008 (10.09) | 12,091 (6.78) |  |
| ≥5 | 28,655 (8.51) | 18,501 (11.66) | 10,154 (5.7) |  |
| Systolic blood pressure, mmHg | 129.12±12.72 | 129.01±12.59 | 129.22±12.83 | <0.001 |
| Fasting serum glucose, mg/dL | 103.52±22.42 | 104.89±23.26 | 102.29±21.58 | <0.001 |
| Total cholesterol, mg/dL | 194.92±34.99 | 187.18±33.21 | 201.81±35.1 | <0.001 |
| Estimated glomerular filtration rate, mL/min/1.73m^2^ |  |  |  | <0.001 |
| <30 | 1,222 (0.36) | 662 (0.42) | 560 (0.31) |  |
| 30-60 | 48,308 (14.34) | 20,564 (12.96) | 27,744 (15.57) |  |
| 60-90 | 222,766 (66.13) | 107,284 (67.62) | 115,482 (64.8) |  |
| ≥90 | 64,583 (19.17) | 30,148 (19.00) | 34,435 (19.32) |  |
| Comorbidities |  |  |  |  |
| Hypertension | 228,485 (67.82) | 104,824 (66.07) | 123,661 (69.39) | <0.001 |
| Diabetes mellitus | 115,440 (34.27) | 54,767 (34.52) | 60,673 (34.04) | 0.001 |
| Dyslipidemia | 184,062 (54.64) | 74,953 (47.24) | 109,109 (61.22) | <0.001 |
| Cancer | 17,891 (5.31) | 10,744 (6.77) | 7,147 (4.01) | <0.001 |
| Renal disease | 63,527 (18.86) | 31,594 (19.91) | 31,933 (17.92) | <0.001 |
| COPD | 23,163 (6.88) | 14,427 (9.09) | 8,736 (4.9) | <0.001 |
| Liver disease | 99,021 (29.39) | 46,977 (29.61) | 52,044 (29.2) | 0.010 |
| OSAS | 176 (0.05) | 131 (0.08) | 45 (0.03) | <0.001 |
| Coronary artery disease | 87,573 (26) | 41,060 (25.88) | 46,513 (26.1) | 0.148 |
| Cardiomyopathy | 809 (0.24) | 410 (0.26) | 399 (0.22) |  |
| Valvular heart disease | 3,016 (0.9) | 1,223 (0.77) | 1,793 (1.01) | <0.001 |
| Charlson Comorbidity Index |  |  |  | <0.001 |
| 0 | 57646 (17.11) | 29,947 (18.88) | 27,699 (15.54) |  |
| 1 | 82664 (24.54) | 39,671 (25) | 42,993 (24.12) |  |
| ≥2 | 196569 (58.35) | 89,040 (56.12) | 107,529 (60.33) |  |

P-values by Student’s t-test and chi-square test. Data are expressed as the mean±SD deviation or n (%).

BMI, body mass index; COPD, chronic obstructive pulmonary disease; OSAS, obstructive sleep apnea syndrome; pASMI, predicted appendicular skeletal muscle mass index; pBFMI, predicted body fat mass index; pLBMI, predicted lean body mass index.

**Supplementary Table 8.** Hazard ratios and 95%CI of heart failure per 1 kg/m^2^ increase in the change in the predicted body composition index (aged 65 and over)

| Variable | Crude |  | Adjusted |  |
| --- | --- | --- | --- | --- |
|  | HR (95%CI) | *P*-value | HR (95%CI) | *P*-value |
| BMI at the baseline period, kg/m^2^ | 1.049 (1.044, 1054) | <0.001 | 1.015 (1.002, 1.028) | 0.024 |
| BMI at the follow-up period, kg/m^2^ | 1.046 (1.042, 1.051) | <0.001 | 1.020 (1.007, 1.003) | 0.003 |
| Household income |  |  |  |  |
| First quintile, lowest | 1 (reference) |  | 1 (reference) |  |
| Second quintile | 1.050 (0.999, 1.104) | 0.057 | 1.030 (0.980, 1.084) | 0.241 |
| Third quintile | 1.013 (0.968, 1.059) | 0.586 | 0.994 (0.950, 1.040) | 0.800 |
| Fourth quintile, highest | 1.031 (0.991, 1.073) | 0.131 | 0.992 (0.954, 1.033) | 0.702 |
| Systolic blood pressure, mmHg | 1.010 (1.009, 1.012) | <.0001 | 1.009 (1.007, 1.010) | <0.001 |
| Fasting serum glucose, mg/dL | 1.001 (1.000, 1.001) | 0.009 | 0.998 (0.997, 0.998) | <0.001 |
| Total cholesterol, mg/dL | 0.998 (0.997, 0.998) | <.0001 | 0.999 (0.999, 1.000) | 0.001 |
| Comorbidities |  |  |  |  |
| Cancer | 0.966 (0.905, 1.031) | 0.304 | 0.838 (0.784, 0.895) | <0.001 |
| Renal disease | 1.054 (1.455, 1.554) | <0.001 | 1.320 (1.277, 1.365) | <0.001 |
| COPD | 1.564 (1.492, 1.640) | <0.001 | 1.334 (1.271, 1.400) | <0.001 |
| Liver disease | 1.186 (1.150, 1.223) | <0.001 | 0.982 (0.952, 1.014) | 0.263 |
| OSAS | 0.211 (0.054, 0.827) | <0.001 | 0.178 (0.045, 0.714) | 0.015 |
| Coronary artery disease | 2.099 (2.038, 2.161) | <0.001 | 1.756 (1.702, 1.811) | <0.001 |
| Cardiomyopathy | 5.325 (4.639, 6.112) | <0.001 | 3.505 (3.051, 4.027) | <0.001 |
| Valvular heart disease | 4.280 (3.952, 4.636) | <0.001 | 3.178 (2.932, 3.444) | <0.001 |
| Charlson Comorbidity Index |  |  |  |  |
| 0 | 1 (reference) |  | 1 (reference) |  |
| 1 | 1.280 (1.213, 1.351) | <0.001 | 1.172 (1.110, 1.238) | <0.001 |
| ≥2 | 1.916 (1.828, 2.008) | <0.001 | 1.477 (1.405, 1.552) | <0.001 |
| Change in the predicted  body composition index |  |  |  |  |
| ΔpASMI, kg/m^2^ | 0.981 (0.966, 0.997) | 0.018 | 0.983 (0.964, 1.002) | 0.080 |
| ΔpBFMI, kg/m^2^ | 0.995 (0.987, 1.003) | 0.185 | 0.999 (0.985, 1.014) | 0.929 |
| ΔpLBMI, kg/m^2^ | 0.987 (0.980, 0.995) | 0.001 | 0.987 (0.976, 0.997) | 0.014 |

The multivariable model was used for the BMI at the baseline and follow-up period, household income, systolic blood pressure, fasting serum glucose, total cholesterol, and Charlson Comorbidity Index.

BMI, body mass index; CI, confidence interval; COPD: Chronic obstructive pulmonary disease; HR, hazard ratio; OSAS: Obstructive sleep apnea syndrome; ΔpASMI, change in predicted appendicular skeletal muscle mass index; ΔpBFMI, change in the predicted body fat mass index; ΔpLBMI, change in the predicted lean body mass index.

**Supplementary Table 9.** Hazard Ratios and 95% Confidence Intervals for Heart Failure per 1 kg/m² Increase in Predicted Body Composition Index by Gender in the 65 and Over Age Group

| Variable | Male |  | Female |  |
| --- | --- | --- | --- | --- |
|  | HR (95%CI) | *P*-value | HR (95%CI) | *P*-value |
| BMI at the baseline period, kg/m^2^ | 1.001 (0.979, 1.024) | 0.904 | 1.016 (1.000, 1.033) | 0.055 |
| BMI at the follow-up period, kg/m^2^ | 1.024 (1.002, 1.047) | 0.033 | 1.021 (1.004, 1.037) | 0.013 |
| Household income |  |  |  |  |
| First quintile, lowest | 1 (reference) |  | 1 (reference) |  |
| Second quintile | 1.030 (0.955, 1.112) | 0.443 | 1.031 (0.964, 1.102) | 0.378 |
| Third quintile | 1.021 (0.954, 1.093) | 0.549 | 0.973 (0.916, 1.033) | 0.372 |
| Fourth quintile, highest | 1.051 (0.990, 1.117) | 0.105 | 0.942 (0.893, 0.993) | 0.027 |
| Systolic blood pressure, mmHg | 1.006 (1.005, 1.008) | <0.001 | 1.010 (1.009, 1.012) | <0.001 |
| Fasting serum glucose, mg/dL | 0.998 (0.997, 0.999) | <0.001 | 0.998 (0.997, 0.999) | 0.001 |
| Total cholesterol, mg/dL | 0.998 (0.998, 0.999) | <0.001 | 0.999 (0.999, 1.000) | 0.015 |
| Comorbidities |  |  |  |  |
| Cancer | 0.882 (0.809, 0.963) | 0.005 | 0.812 (0.733, 0.900) | <0.001 |
| Renal disease | 1.323 (1.259, 1.391) | <0.001 | 1.328 (1.269, 1.389) | <0.001 |
| COPD | 1.268 (1.186, 1.355) | <0.001 | 1.474 (1.374, 1.583) | <0.001 |
| Liver disease | 1.003 (0.956, 1.052) | 0.902 | 0.972 (0.932, 1.013) | 0.181 |
| OSAS | 0.131 (0.018, 0.928) | 0.042 | 0.304 (0.043, 2.155) | 0.233 |
| Coronary artery disease | 1.799 (1.716, 1.886) | <0.001 | 1.721 (1.652, 1.793) | <0.001 |
| Cardiomyopathy | 4.058 (3.352, 4.912) | <0.001 | 3.041 (2.484, 3.722) | <0.001 |
| Valvular heart disease | 3.213 (2.831, 3.646) | <0.001 | 3.124 (2.814, 3.469) | <0.001 |
| Charlson Comorbidity Index |  |  |  |  |
| 0 | 1 (reference) |  | 1 (reference) |  |
| 1 | 1.106 (1.022, 1.198) | 0.013 | 1.213 (1.125, 1.307) | <.0001 |
| ≥2 | 1.406 (1.306, 1.513) | <.0001 | 1.484 (1.385, 1.589) | <.0001 |
| Change in the predicted  body composition index |  |  |  |  |
| ΔpASMI, kg/m^2^ | 0.982 (0.956, 1.008) | 0.176 | 0.973 (0.945, 1.002) | 0.068 |
| ΔpBFMI, kg/m^2^ | 1.016 (0.999, 1.034) | 0.065 | 0.968 (0.940, 0.996) | 0.025 |
| ΔpLBMI, kg/m^2^ | 0.991 (0.977, 1.005) | 0.189 | 0.972 (0.948, 0.997) | 0.030 |

The multivariable model was used for the BMI at the baseline and follow-up period, household income, systolic blood pressure, fasting serum glucose, total cholesterol, and Charlson Comorbidity Index.

BMI, body mass index; CI, confidence interval; COPD: Chronic obstructive pulmonary disease; HR, hazard ratio; OSAS: Obstructive sleep apnea syndrome; ΔpASMI, change in predicted appendicular skeletal muscle mass index; ΔpBFMI, change in the predicted body fat mass index; ΔpLBMI, change in the predicted lean body mass index.
