# Supplementary material for: Association between changes in predicted body composition and occurrence of heart failure: a nationwide population study
Source: Front Endocrinol (Lausanne). 2023 Oct 23;14:1210371. doi: 10.3389/fendo.2023.1210371 (PMC10627176; doi:10.3389/fendo.2023.1210371)
Supplement: Supplementary file 2 [file DataSheet_2.docx]

**Supplementary Method 1.** Anthropometric prediction equations for lean body mass, appendicular skeletal muscle mass, and body fat mass.

| Prediction equations for men^a^ | | |
| --- | --- | --- |
| LBM (kg) | -0.296 + 0.012*age (years) + 0.134*height (cm) + 0.675*weight (kg) - 0.201*waist (cm) - 0.249*serum creatinine (mg/dL) + 0.270*moderate physical activity + 0.924*vigorous physical activity – 0.559*past smoker + 0.234*current smoker - 0.046*moderate drinker + 0.324*heavy drinker | R^2^=0.86  SEE=2.67 |
| ASM (kg) | -2.236 - 0.011*age (years) + 0.081*height (cm) + 0.324*weight (kg) - 0.121*waist (cm) – 0.008*serum creatinine (mg/dL) + 0.200*moderate physical activity + 0.587*vigorous physical activity – 0.195*past smoker – 0.016*current smoker + 0.004*moderate drinker + 0.151*heavy drinker | R^2^=0.81  SEE=1.56 |
| BFM (kg) | 0.561 - 0.012*age (years) - 0.133*height (cm) + 0.310*weight (kg) + 0.199*waist (cm) + 0.253*serum creatinine (mg/dL) - 0.247*moderate physical activity - 0.878*vigorous physical activity + 0.708*past smoker - 0.275*current smoker + 0.077*moderate drinker - 0.291*heavy drinker | R^2^=0.75  SEE=2.70 |
| Prediction equations for women^a^ | | |
| LBM (kg) | -11.941 + 0.015*age (years) + 0.171*height (cm) + 0.457*weight (kg) - 0.060*waist (cm) + 0.428*serum creatinine (mg/dL) + 0.181*moderate physical activity + 0.654*vigorous physical activity – 0.254*past smoker + 0.221*current smoker + 0.209*moderate drinker + 0.126*heavy drinker | R^2^=0.79  SEE=2.18 |
| ASM (kg) | -8.447 + 0.002*age (years) + 0.091*height (cm) + 0.203*weight (kg) - 0.034*waist (cm) + 0.539*serum creatinine (mg/dL) + 0.103*moderate physical activity + 0.362*vigorous physical activity – 0.203*past smoker + 0.000*current smoker + 0.103*moderate drinker + 0.031*heavy drinker | R^2^=0.72  SEE=1.20 |
| BFM (kg) | 12.269 - 0.014*age (years) - 0.172*height (cm) + 0.530*weight (kg) + 0.058*waist (cm) – 0.314*serum creatinine (mg/dL) - 0.123*moderate physical activity - 0.541*vigorous physical activity + 0.261*past smoker - 0.242*current smoker - 0.218*moderate drinker - 0.182*heavy drinker | R^2^=0.83  SEE=2.24 |

^a^ The prediction equations were derived and validated using the Korean National Health and Nutrition Examination Survey 2008–2011.

Physical activity, smoking, and alcohol intake are binary variables (yes=1; no=0) as follows:

Moderate physical activity: those who engage in 600-2999 METs-min/week of physical activity;

Vigorous physical activity: those who engage in ≥3000 METs-min/week of physical activity;

Moderate drinker: drinking alcohol less than 14 drinks per week (men), or less than 7 drinks per week (women);

Heavy drinker: drinking alcohol 14 drinks or more per week (men), or 7 drinks or more per week (women).

LBM, Lean body mass; ASM, Appendicular skeletal muscle mass; BFM, Body fat mass; SEE standard error of estimate; MET, Metabolic Equivalent Task.

**Supplementary Methods 2.** Definition of covariates

**Smoking status**

Definition and measurement of variables like usual smoking status were obtained by questionnaire in the health examination program. Smoking status was used to categorize participants into three groups: none, former smoker, and current smoker. Current smoker was classified according to the WHO definition as a person who has smoked more than five packs (100 cigarettes) in a lifetime and smoked daily or occasionally for the last 28 days. Former smoker was defined as a person who had smoked more than 100 cigarettes in a lifetime and had not smoked in the last 28 days (1).

**Alcohol intake**

Definition and measurement of variables like usual alcohol consumption were obtained by questionnaire in the health examination program. Alcohol consumption was categorized into four groups: 0, 1-2, 3-4, or ≥5 times per week. For anthropometric prediction equations, differentiation between moderate and heavy drinker was based on whether a patient usually takes more than 14 drinks/7 drinks per week for men/women. The drinks were calculated by multiplying the average drinking frequency per week by the number of drinks per occasion.

**Physical activity**

Physical activity was assessed using the Korean version of the International Physical Activity Questionnaire-short form. Physical activity was categorized into four groups: 0, 1-2, 3-4, or ≥5 times per week. For anthropometric prediction equations, we created composite physical activity based on Metabolic Equivalent Task (MET)-minutes/week (walking: 3.3 METs; moderate physical activity: 4.0 METs; vigorous physical activity: 8.0 METs), which was categorized as follows based on total physical activity metabolic equivalents: low (< 600 METs), moderate (600–2,999 METs), and vigorous (≥ 3,000 METs) (2, 3).

**Hypertension**

Hypertension was defined as using at least one claim of ICD-10 code(I10-15) with the prescription of an anti-hypertensive agent, claims of ICD-10 code(I10-15) more than two times, a systolic blood pressure of ≥140 mmHg and a diastolic blood pressure of ≥90 mmHg or positive checking in self-report questionnaire on hypertension in the health examination program.

**Diabetes mellitus**

Diabetes mellitus was as defined using at least one claim of ICD-10 code(E11-14) with the prescription of a anti-diabetic agent, claims of ICD-10 code(E11-14) more than two times, fasting serum glucose concentration of ≥7.0 mmol/L or positive checking in self-report questionnaire on diabetes mellitus in the health examination program.

**Dyslipidemia**

Dyslipidemia was defined as using at least one claim of ICD-10 code(E78) with the prescription of an anti-dyslipidemic agent, claims of ICD-10 code(E78) more than two times or total cholesterol level of ≥240 mg/dL.

**Cancer**

Cancer was defined as using claims of ICD-10 code(C00–C97) more than two times with cancer specific deductible code (V027, V193-4) from the Health Insurance Review and Assessment Service.

**Renal disease**

Renal disease was defined as using claims of ICD–10 codes (N17-19, I12-13, E08.2, E10.2, E11.2, E13.2) more than two times or estimated glomerular filtration rate (eGFR) of <60 mL/min/1.73m^2^.

**Charlson comorbidity index**

The Charlson comorbidity index score was calculated for each subject based on diseases diagnosed before index date and divided into three groups (0, 1, and ≥2 scores) (4).

**References**

1. Lee KH, Lee CM, Kwon HT, Oh S-W. Relationship between Obesity and Smoking in Korean Men: Data Analyses from the Third and Fourth Korea National Health and Nutrition Examination Surveys (KNHANES). JKSRNT. 2010;1(2):115-23.

2. Oh JY, Yang YJ, Kim BS, Kang JH. Validity and Reliability of Korean Version of International Physical Activity Questionnaire (IPAQ) Short Form. J Korean Acad Fam Med. 2007;28(7):532-41.

3. Do-Hyun K, Eun-Joon L, Ji-Yeon L, Duk-Chul L. The association and the characteristics of the smoking status and differences in physical activity level in Korean adults: The Sixth Korea National Health and Nutrition Examination Survey (KNHANES VI-1), 2013. KJFP. 2015;5(3):510-6.

4. Kim KH. Comorbidity Adjustment in Health Insurance Claim Database. Health Policy and Management. 2016;26(1):71-8.
