# Supplementary figures and images for: Association between changes in predicted body composition and occurrence of heart failure: a nationwide population study
Source: Front Endocrinol (Lausanne). 2023 Oct 23;14:1210371. doi: 10.3389/fendo.2023.1210371 (PMC10627176; doi:10.3389/fendo.2023.1210371)

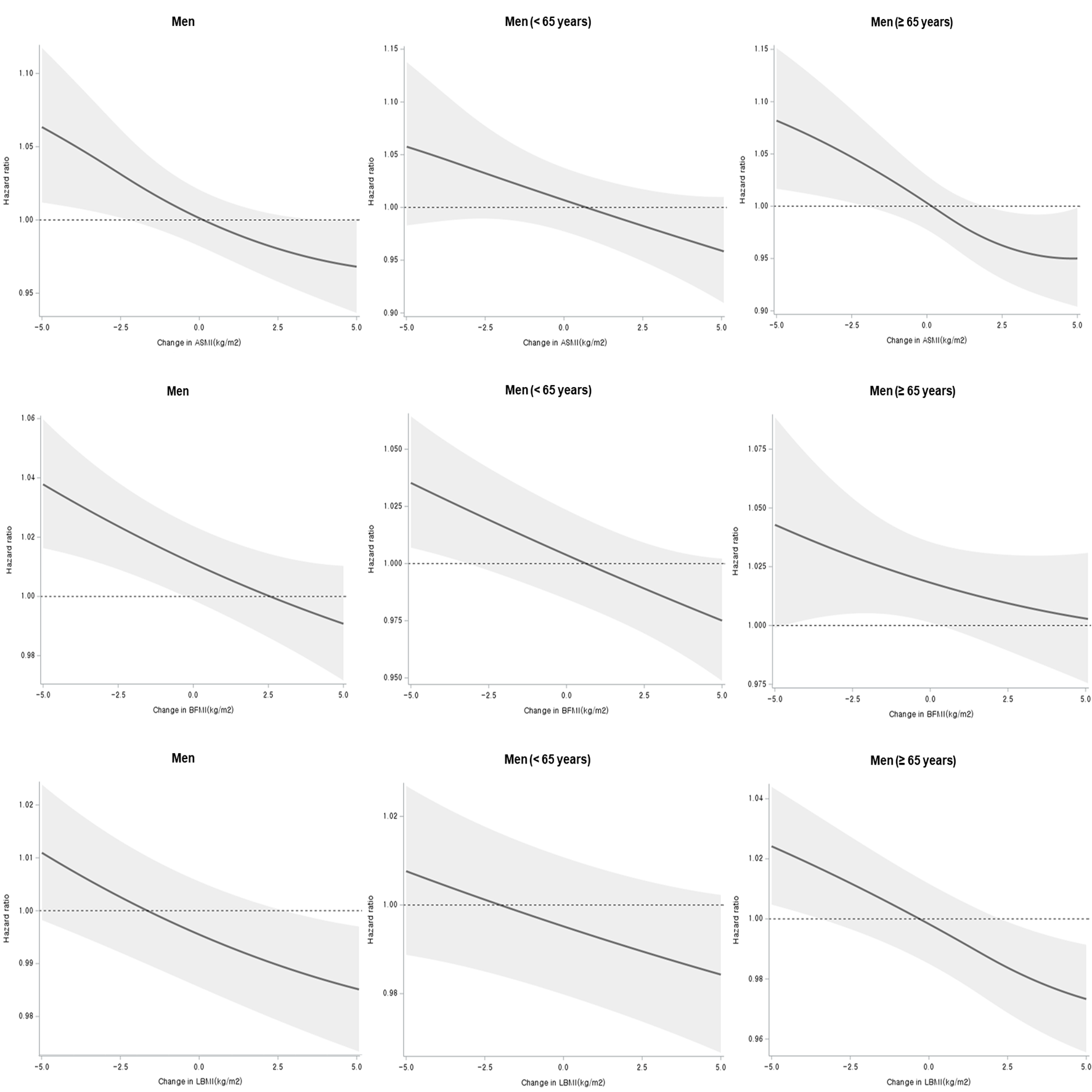

Supplement: Supplementary file 3 [file Image_1.tif]

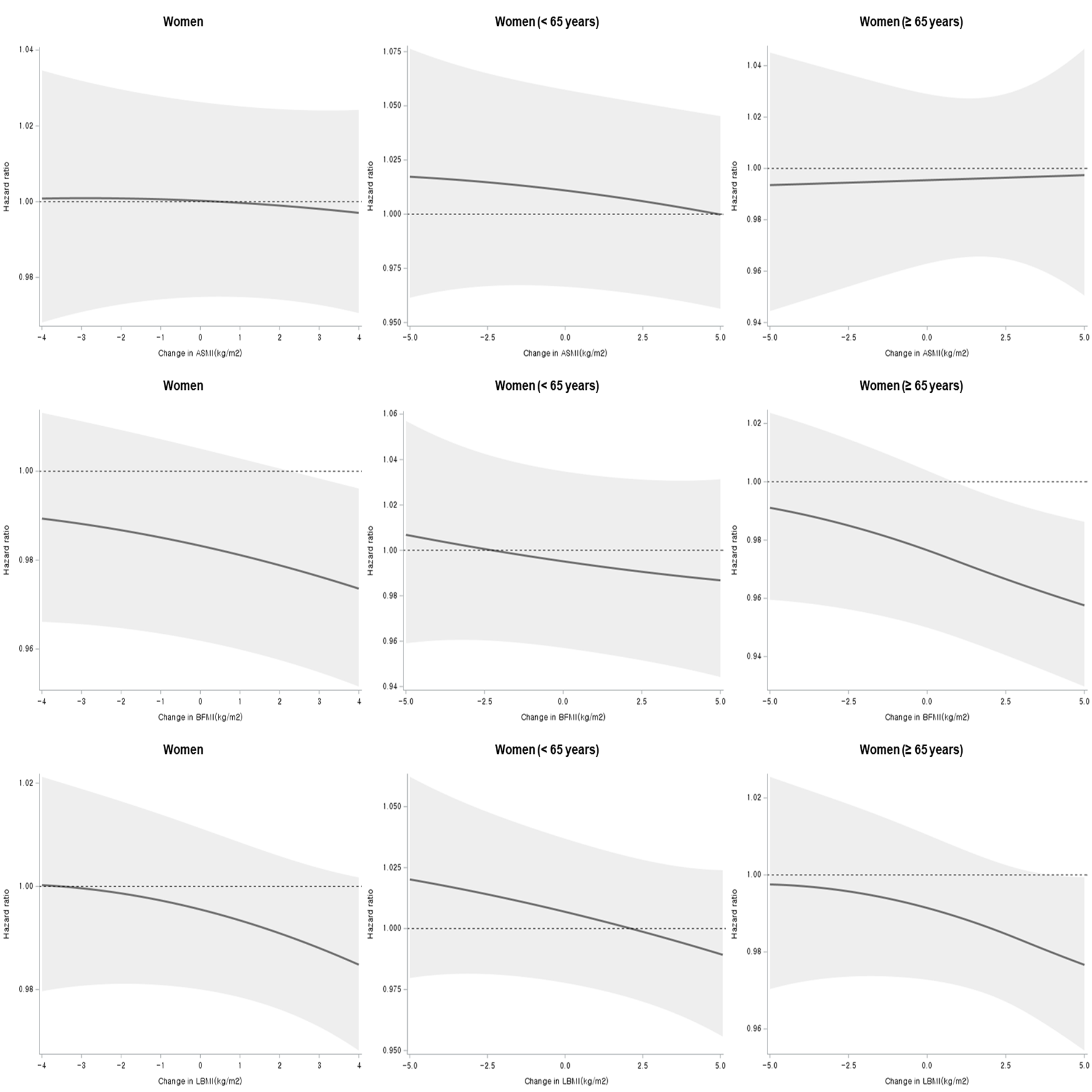

Supplement: Supplementary file 4 [file Image_2.tif]
